# Transcriptome Profiling of Eutopic and Ectopic Endometrial Stromal Cells in Women with Endometriosis Based on High-Throughput Sequencing

**DOI:** 10.3390/biomedicines10102432

**Published:** 2022-09-29

**Authors:** Chih-Chieh Chen, Yung-Che Chou, Chia-Yi Hsu, Eing-Mei Tsai, Tze-Kiong Er

**Affiliations:** 1 Institute of Medical Science and Technology, National Sun Yat-sen University, Kaohsiung 804, Taiwan; 2 Division of Laboratory Medicine, Asia University Hospital, Asia University, Taichung 413, Taiwan; 3 Department of Obstetrics and Gynecology, Kaohsiung Medical University Hospital, Kaohsiung 807, Taiwan; 4 Department of Medical Laboratory Science and Biotechnology, Asia University, Taichung 413, Taiwan

**Keywords:** endometriosis, transcriptome, RNA sequencing, and gene ontology analysis

## Abstract

Endometriosis is a common gynecological disease that affects approximately 5–10% of reproductive-aged women. However, the etiology and pathophysiology of endometriosis are currently unclear. The objective of this study was to identify a potential pathogenic gene of endometriosis using RNA sequencing (RNA-seq) analysis. Human endometrial stromal cells were isolated from four patients receiving surgical treatment for endometriosis during laparoscopic surgery, and RNA-seq was used to examine differentially expressed genes (DEGs) in eutopic and ectopic endometrial stromal cells. The functional significance of the differentially expressed genes was analyzed using Gene Ontology (GO) and Kyoto Encyclopedia of Genes and Genomes (KEGG) pathway enrichment analyses. A total of 1309 upregulated and 663 downregulated genes were identified through the analysis of the transcriptomes of eutopic and ectopic endometrial stromal cells. Furthermore, KEGG analysis indicated that these DEGs were mainly enriched in the PI3K-Akt signaling pathway, cytokine–cytokine receptor interaction, and MAPK signaling pathway. Our study identified differential gene expression in eutopic as compared to ectopic endometrial tissue stromal cells. We strongly believe that our findings can bring new insights into the underlying mechanisms of endometriosis. However, future research is necessary to clarify the roles of the identified genes.

## 1. Introduction

Endometriosis is a benign gynecological condition that affects approximately 5–10% of reproductive-aged women, leading to multiple symptoms [1]. Endometriosis is defined as the presence of endometrial glands and stroma-like tissue outside the uterus [2]. In Taiwan, the condition occurs in approximately 8.9% of the general population [3]. Endometriosis is a complex ailment with a multifactorial pathogenesis [4]. Therefore, it is rational to suggest that potential alterations and expression changes in several genes may initiate the development of the disease [5]. 

Even though endometriosis has been studied for a long time, there are still no practical biomarkers, and endometriosis-associated genes and pathways remain unclear. Until now, the identification of this disease has been achieved through laparoscopy. However, surgical interventions can cause complications, including hemorrhage, infection, and adhesion formation [6]. Therefore, noninvasive biomarkers for the early stages of endometriosis are urgently needed to enable early treatment [7]. One study showed that VEGF-C can be used as a diagnostic biomarker for endometriosis [8]. Until now, it has not been possible to predict the presence of endometriosis based on clinical symptoms, clinical examination, diagnostic imaging techniques, or blood testing. 

Next-generation sequencing (NGS) has been widely used in whole-genome sequencing, targeted DNA sequencing, RNA sequencing (RNA-seq), and epigenomics [9]. Our previous review article indicated that NGS is a promising technology and may provide new clues in the pathogenesis of endometriosis [10]. A study demonstrated an overview of differentially expressed lncRNAs, circRNAs, and mRNAs in endometriosis, and the author suggested that lncRNAs and circRNAs exerted important functions in the pathogenesis of endometriosis [11]. Additionally, through single-cell transcriptomic analysis, Ma J et al. demonstrated that the development of the endometriosis is due to an imbalanced immune environment in endometriosis lesions [12]. Besides, Zou G et al. revealed that immune dysfunction occurs in the peritoneal fluid of endometriosis using single-cell RNA sequencing [13]. They concluded that immune dysfunction may play an important role in the pathology of endometriosis. Moreover, Tan Y et al. analyzed the transcriptome of endometrium and endometriotic lesions using single-cell RNA sequencing and provided a landscape of the endometriosis microenvironment [14]. In general, evaluating transcriptome datasets facilitates the assessment of overall gene functions and structures to investigate the molecular signatures predictive of certain diseases.

In the current study, we sought to determine differentially expressed genes (DEGs) related to endometriosis using RNA-seq of cultured endometrial stromal cells from paired samples from the same woman. Through transcriptomic profiling, we aimed to elucidate potential novel genes and significant pathways that are involved in the development of endometriosis. We hope that our findings will identify biomarkers for the early diagnosis of endometriosis.

## 2. Materials and Methods

### 2.1. Isolation of Eutopic and Ectopic Endometrial Stromal Cells

Human endometrial stromal cells were separated from four patients (aged 31–45 years) undergoing surgery for the treatment of endometriosis in the Department of Obstetrics and Gynecology of Kaohsiung Medical University Hospital of Kaohsiung Medical University in Kaohsiung, Taiwan (Table S1). The utilization of these tissues was approved by the Institutional Review Board (KMUHIRB-20140031), and written informed consent was obtained from each patient. None of the patients had received previous treatment for endometriosis. The patients were of reproductive age and had a surgically and histologically confirmed diagnosis of stage III or IV endometriosis. The patients had a normal menstrual cycle and lack of chronic diseases. All specimens were determined as being in the proliferative phases according to pathologic results and/or menstrual cycles. The revised American Society for Reproductive Medicine classification was used to determine the disease stage. All patients underwent laparoscopic confirmation for the diagnosis of endometriosis, and none with endometrial pathologic conditions were found. Additionally, the patients had not previously received any treatment for endometriosis.

In the current study, eutopic and ectopic endometrial stromal cells were isolated using the previously described technique [15,16]. Ectopic endometrial stromal cells were processed from endometrioma cysts of endometriosis patients. On the other hand, eutopic endometrial stromal cells were processed from the eutopic endometrium of those patients. Phosphate-buffered saline (PBS) was used to wash the tissues. Then, type IV collagenase (2 mg/mL) and DNase I (100 μg/mL) in PBS were used to digest the tissues and with shacking at 100 rpm for 60 min at 37 °C. First, stromal cells were isolated from epithelial cells by filtration through nylon mesh with a pore size of 70 μm and then a pore size pf 40 μm. Then, the filtered cells were allowed to attach for 30 min in a T-75 flask. After that, PBS was used to remove tissue debris, blood cells, and epithelial cells. Finally, stromal cells were cultured in DMEM/F12 medium with 10% FBS in a humidified atmosphere with 5% CO2 at 37 °C. We performed immunofluorescence staining of vimentin (a stromal cell cytoskeletal marker), which was used as a positive marker to confirm the purity of stromal cells. The culture medium was replaced with serum-free medium when the subcultured cells reached 70% confluence within 24 h. The cells that originated from the 5th to 6th passages were used in this study. Finally, the endometrial stromal cells were stocked and frozen for further experiments. 

### 2.2. RNA Isolation and Quality Assessment

TRIzol Reagent (Sigma-Aldrich, St. Louis, MO, USA.) was applied to extract the total RNA according to the manufacturer’s instructions. An ND-1000 spectrophotometer (Nanodrop Technology, Wilmington, USA) was used to quantify purified RNA at OD260 nm. Additionally, a Bioanalyzer 2100 (Agilent Technologies, Santa Clara, CA, USA) combined with an RNA 6000 LabChip kit (Agilent Technologies, Santa Clara, CA, USA) was used to qualitatively purify RNA and RNA integrity.

### 2.3. RNA Sequencing

The samples were sent to Welgene Biotechnology Company (Welgene, Taipei, Taiwan) for RNA-seq. All procedures for RNA-seq were performed according to the instructions from the manufacturer, Illumina. The RNA library was constructed using the the Agilent’s SureSelect Strand Specific RNA Library Preparation Kit, accompanied by AMPure XP Beads size selection. Paired-end sequences with a read length of 150 nucleotides were determined by the Illumina’s sequencing-by-synthesis technology. Welgene’s pipeline was applied to generate the sequencing data according to the manufacturer’s instructions (Illumina). The raw reads were trimmed for qualified reads. The lower quality bases were removed using Trimmomatic (version 0.36) [17] at the same time. After that, the HISAT2 alignment tool was used to align the qualified reads to the reference human genome as previously described [18]. The gene expression level was calculated in terms of fragments per kilobase of transcript per million mapped reads (TPM). Differential expression analysis was performed using StringTie (StringTir v2.1.4) and DEseq2 (v1.28.1) with genome bias detection/correction as well as Welgene in-house methods were used to analyze the differential expression between eutopic and ectopic endometrial stromal cells. If the genes exhibited low expression levels (<0.3 TPM value), they were excluded, while those with false discovery rate (FDR) value ≤0.05 and ≥ 2-fold changes were considered significant.

### 2.4. Functional Analysis Using Different Bioinformatics Tools

R packages clusterProfiler v3.6 which combines with the Gene Ontology (GO) and Kyoto Encyclopedia of Genes and Genomes (KEGG) databases to elucidate biological functions, was used to examine the functional roles of DEGs. To gain more information, GO was used to provide three classifications: biological processes, molecular functions, and cellular components. Biological analyses were conducted using the R packages clusterProfiler v3.6 to elucidate the function of DEGs. The GO functional enrichment assay and KEGG pathway enrichment assay were calculated to be statistically significant when adjusted *p* values < 0.05, respectively. Here, hierarchical clustering analysis was undertaken by complete linkage, and the Euclidean distance was used as a measure of similarity to display the expression patterns of DEGs. Typically, the fold change and *p* value criteria were greater than or equal to 2 and *p* < 0.05. All data in this study and DEG visualization were processed through the R project for statistical computing (www.r-project.org) (accessed on 25 September 2022).

Furthermore, the cnetplot was constructed using the R packages clusterProfiler v3.6 and ‘ReactomePA’, and a principal component analysis (PCA) plot was generated using the FactoMineR (https://cran.r-project.org/web/packages/FactoMineR/) (accessed on 25 September 2022) and factoextra (https://cran.r-project.org/web/packages/factoextra/) (accessed on 25 September 2022) packages in R with TPM values.

## 3. Results

### 3.1. mRNA Filtering and Mapping

The mRNA sequencing produced 594,765,518 reads, with average values of 85,698,796 reads and 62,994,816 reads per sample in the eutopic and ectopic endometrial stromal cells group, respectively. A fastQC quality test showed that 573,965,248 (96.5%) reads had a Q-score ≥ 20, which were then considered for further analysis. Approximately, 97.07% of these reads were aligned to hg38.

### 3.2. GO Analysis

**Figure 1:**
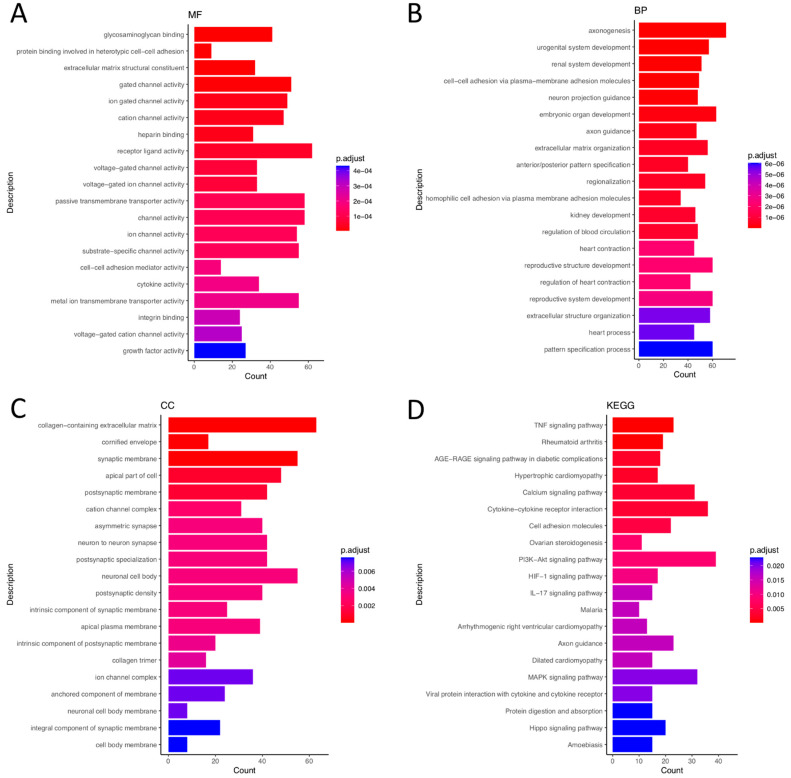
The GO terms of differential expressed genes in eutopic endometrial stromal cells and ectopic endometrial stromal cells. (**A**) GO: MF (Molecular Function); (**B**) GO: BP (Biological Process); (**C**) GO: CC (Cellular Component); (**D**) KEGG Pathway.

The GO enrichment analysis consisted of three items: (1) Biological processes: urogenital system development (GO:0001655), axonogenesis (GO:0007409), cell–cell adhesion via plasma membrane adhesion molecules (GO:0098742), etc. (2) Molecular functions: glycosaminoglycan binding (GO:0005539), protein binding involved in heterotypic cell–cell adhesion (GO:0086080), extracellular matrix structural constituent (GO:0005201), etc. (3) Cellular components: collagen-containing extracellular matrix (GO:0062023), cornified envelope (GO:0001533), synaptic membrane (GO:0097060), etc (Figure 1A–D). Furthermore, the cnetplot diagram shows the links between genes and biological processes by using GO as networks, including cell–cell adhesion via plasma membrane adhesion molecules, axonogenesis, neuron projection guidance, urogenital system development, and renal system development (Figure S1).

### 3.3. Analysis of Differentially Expressed Genes

**Figure 2:**
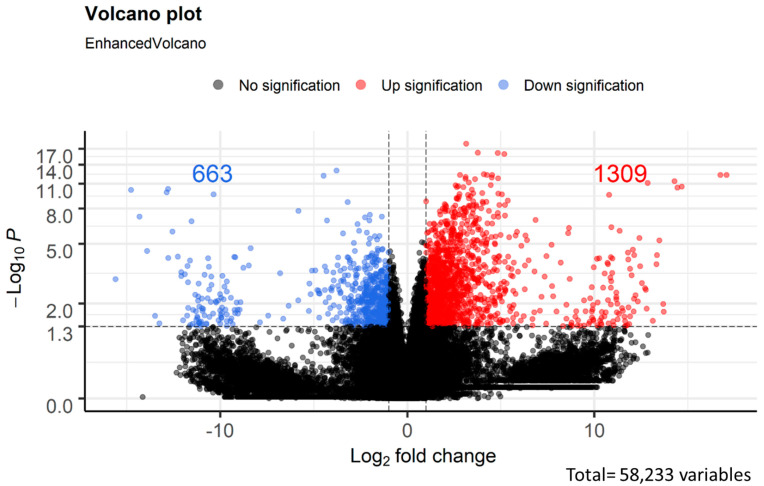
Volcano plot of differential gene expression.

TPM was used to quantify gene expression. On the other hand, the expression of abundance was calculated and represented as volcano plots (Figure 2). In this study, a total of 1972 DEGs were analyzed, and 1309 and 663 were upregulated and downregulated, respectively. The top ten differentially expressed genes are summarized in Table S2. The top four genes were *SEC14L4* (log_2_FC = 10.9), *SNAI3* (log_2_FC = 10.3), *MYL3* (log_2_FC = −14.3), and *GLRA4* (log_2_FC = −10.6).

### 3.4. Hierarchical Clustering Analysis

**Figure 3:**
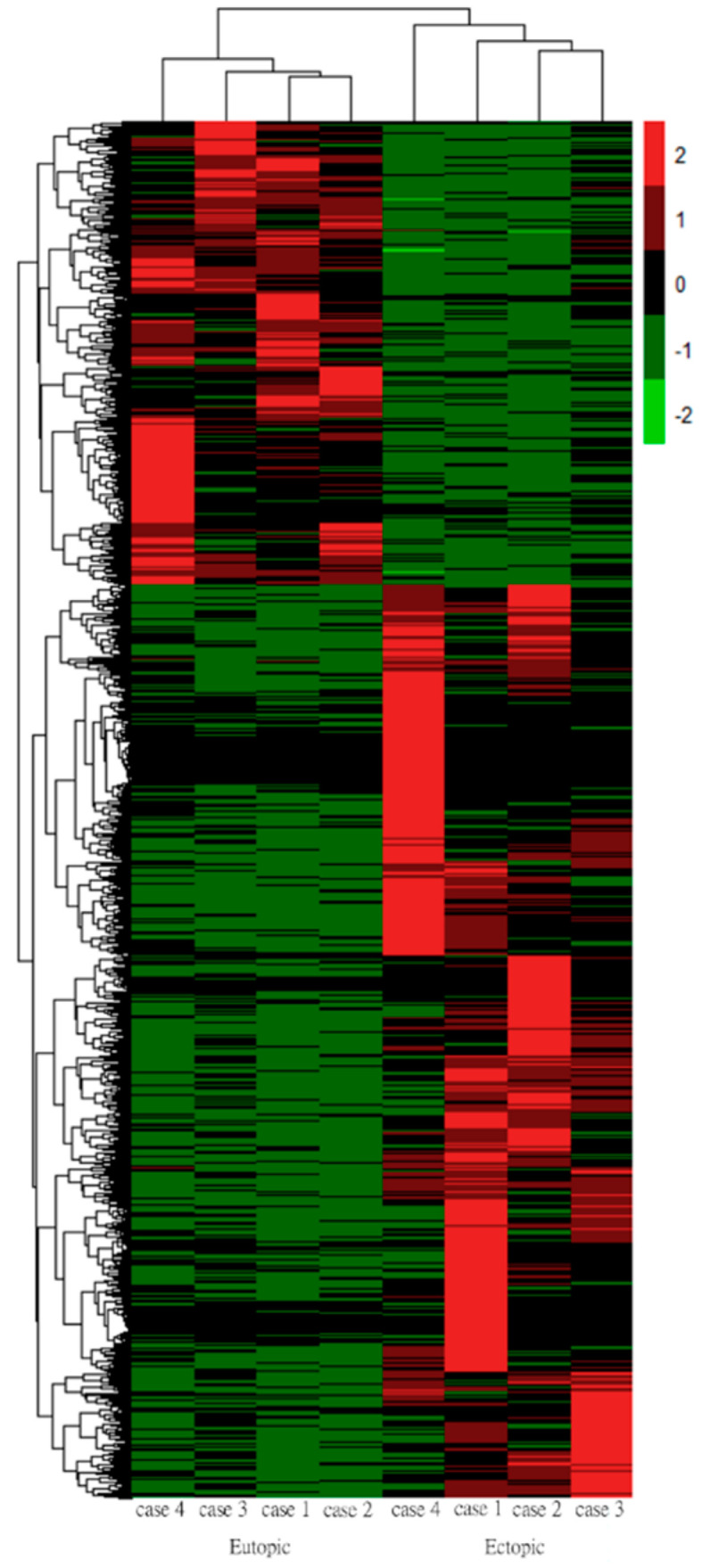
Heatmap of each patient’s gene expression. Rows represent each sample, and columns represent different genes. The gene expression differences are highlighted in green (downregulation) and red (upregulation).

Euclidean distance with average linkage was applied to construct a heatmap for DEGs between eutopic and ectopic endometrial stromal cells. The heatmap showed that the differences between the eutopic and ectopic endometrial stromal cells were statistically significant. The Z-score-centered log2-transformed gene in each sample is shown by using a color scale; the gene expression differences highlighted in green represent downregulation, and red represents upregulation (Figure 3).

### 3.5. KEGG Analysis

**Figure 4:**
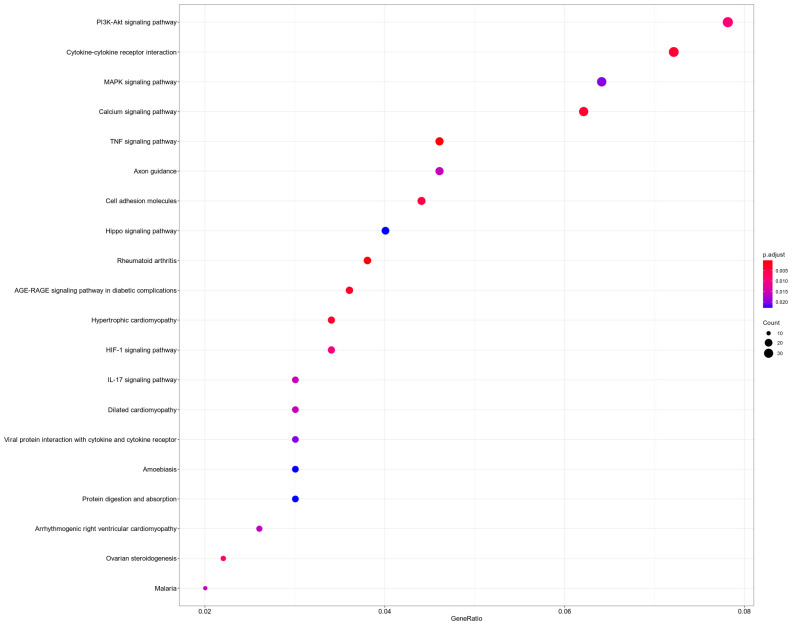
Kyoto Encyclopedia of Genes and Genomes enrichment of differentially expressed genes. Top 20 signaling pathways derived from KEGG signaling pathway enrichment. The point size indicates the number of differentially expressed genes in that gene class, and the color indicates the enrichment effect. The abscissa is the gene ratio, and the larger the number, the greater the enrichment degree.

A total of 1972 DEGs were mapped to KEGG pathways. Of the pathway annotations and analyses, 28 were significantly different (*p* < 0.05). The most differentially activated pathways included the PI3K-Akt signaling pathway, cytokine–cytokine receptor interaction, and MAPK signaling pathway (Figure 4). The lists of genes in each category are summarized in Table S3.

### 3.6. PCA Plot of RNA-seq Data

The transcriptomes of endometrial stromal cells in the dataset should be alike because the cells were separated from primary cultures and an expert simultaneously conducted RNA-seq experiments. The gene expression (TPM) PCA plot was used to determine the characteristics of the RNA-seq data (Figure S2). Based on our findings, we demonstrated that the RNA-seq data of the samples were identical to the other samples within the same group (eutopic or ectopic).

## 4. Discussion

Today, transcriptome sequencing by NGS contributes to gene expression profiling studies, together with the identification of mutations, sequence aberrations, alternative splicing, and RNA editing [19]. One of the most common applications of RNA-seq is to analyze differential gene expression [20]. In addition, high-throughput RNA-seq technology has been utilized to investigate the transcriptome [21]. In addition, RNA-seq is used to identify DEGs to discover the intracellular molecular pathways and networks [22]. More importantly, differential gene expression analysis allows for the identification of potential biomarkers [23]. Recently, Adamyan L et al. identified five specific genes downregulated in the endometrial samples and endometriotic lesions of patients with endometriosis using RNA-seq [24]. Moreover, the findings of Zhao LY et al. indicate that five genes may act as novel biomarkers in patients with endometriosis [25].

Our findings provide the transcriptomic profiling of the human endometrial stromal cells obtained from women with endometriosis using RNA-seq. As mentioned above, endometriosis is a complex and a multifactorial disease; thus, abnormal up- or downregulation of one gene cannot sufficiently display complicated transcriptome alterations in the development of endometriosis. In the current study, GO and KEGG pathway annotations were applied to investigate the transcriptome data of cultured endometrial stromal cells from paired samples from the same woman. It is worth noting that stromal cells are the most abundant cell type in the endometrium [26]. However, only a few studies have been conducted using pure cell populations for elucidating gene expression in endometriotic lesions. Previously, Rekker K et al. indicated that the use of isolated stromal cells facilitates the determination of gene expression levels and avoids interference by other cell types [27]. In this study, KEGG analysis demonstrated that DEGs were mainly involved in the three pathways as shown in Figure 4: the PI3K-Akt signaling pathway, cytokine–cytokine receptor interaction, and MAPK signaling pathway.

A study showed that not only the dysregulation of signaling pathways but also cytokines may trigger the formation of endometriosis [28]. The phosphatidylinositol-3 kinase (PI3K)/protein kinase B (AKT) signaling pathway is a pivotal intracellular signal transduction pathway, and the function of this pathway is the maintenance of the physiological functions [29]. In vitro evidence indicates that dysregulation of the PI3K/mTOR pathway decreases the proliferation of endometriotic epithelial and stromal cells [30]. Recently, Madanes D et al. emphasized the importance of the PI3K/AKT signaling pathway and demonstrated clear differences between the stages of endometriosis [31]. In addition, Yin X et al. showed that overactivation of the PI3K/AKT signaling pathway leads to the reduced expression of the decidua-specific genes in endometriotic stromal cells [32]. Taken together, the findings of previous studies highlighted the critical role of the PI3K/AKT pathway in the pathogenesis of endometriosis.

Cytokines and chemokines serve in pivotal roles in the pathological processes of endometriosis. Additionally, the dysregulation of several specific pathways involving leukocyte activation and cytokine–cytokine receptor interactions in endometriosis has also been demonstrated [33]. In addition, Ahn SH et al. showed that immune-inflammation genes in endometriosis patients were differentially expressed [34]. Differential expression of anti-inflammatory cytokines has also been demonstrated in women with endometriosis. These anti-inflammatory cytokines also play important roles in the development of endometriosis by promoting cell survival, invasion, angiogenesis, and the immune escape of endometrial fragments [35]. Moreover, Li S et al. demonstrated that IL-6 and INF-γ are involved in the pathological processes of endometriosis [36]. Previously, Sun JL et al. showed that IL-10 may be associated with the development of endometriosis [37]. In addition, the authors demonstrated that local plasmacytoid dendritic cell-secreted IL-10 induces endometriogenesis through pathological angiogenesis during the initiation of the disease stage. Overall, cytokine–cytokine receptor interaction plays a crucial role in the pathogenesis of endometriosis.

Mitogen-activated protein kinase (MAPK) cascades have been demonstrated as playing a key role in transducing extracellular signals to cellular responses. In mammalian cells, MAPK pathways can mediate and integrate signals from a various range of stimuli and induce appropriate physiological responses [38]. The MAPK signaling pathway has multiple roles in creating the microenvironment and promoting patients’ survival of endometriosis. Patients with endometriosis may worsen due to abnormal MAPK activation [39]. Recently, Liu ZH et al. demonstrated that the overexpression of TGF-β enhances the migration and invasion of ectopic endometrial stromal cells via the ERK/MAPK signaling pathway [40]. Meanwhile, a genome-wide association study showed that integrin-mediated MAPK activation plays an important role in the formation of endometriotic lesions [41]. In summary, these findings provide evidence of the crucial roles of MAPK pathways in the pathogenesis of endometriosis.

Through transcriptomic profiling, we determined potential pathways that are involved in the pathogenesis of endometriosis. In summary, transcriptomes from four paired samples were evaluated, and our findings revealed 1309 upregulated and 663 downregulated genes. These DEGs were mostly involved in three pathways: the PI3K-Akt signaling pathway, cytokine–cytokine receptor interaction, and the MAPK signaling pathway. These pathways may represent promising biomarkers and therapeutic targets for endometriosis. However, the main drawback of the current study is its small sample size. Future studies should aim to overcome the limitations of this work by expanding the sample size; in addition, future research is needed to understand the biological functions of differentially expressed pathways in the pathogenesis of endometriosis. Furthermore, the purity of the isolated cells should be considered before performing RNA-Seq. We strongly believe that our study will provide new insights into the underlying mechanisms of endometriosis and help to discover potential biomarkers for early diagnosis.

## 5. Conclusions

We strongly believe that our study will provide new insights into the pathogenesis of endometriosis and help to discover potential biomarkers for diagnosis.

## Data Availability

All data associated with this study are presented in the paper.

## Supplementary Materials

The following supporting information can be downloaded at: https://www.mdpi.com/article/10.3390/biomedicines10102432/s1. Table S1: The clinical characteristics of the four patients with endometriosis; Table S2: Top 10 differentially expressed upregulated and downregulated mRNAs with the highest fold change; Table S3: KEGG analysis showed that the DEGs were mostly involved in three pathways: the PI3K-Akt signaling pathway, cytokine-cytokine receptor interaction, and MAPK signaling pathway; Figure S1: The genes associated with the five GO biological processes terms are illustrated by the cnetplot; Figure S2: PCA plot of RNA-seq data show the characteristics of samples according to gene expression (TPM) levels. Each dot indicates a sample.
