# Supplementary material for: Transcriptome Profiling of Eutopic and Ectopic Endometrial Stromal Cells in Women with Endometriosis Based on High-Throughput Sequencing
Source: Biomedicines. 2022 Sep 29;10(10):2432. doi: 10.3390/biomedicines10102432 (PMC9598494; doi:10.3390/biomedicines10102432)
Supplement: Supplementary file 1 [file biomedicines-10-02432-s001.zip › biomedicines-1931278-supplementary.pdf]

Supplementary Materials:

Table S1. The clinical characteristics of the four patients with endometriosis.

Case no.	Age, years	Stage	Sample Name	Type
1	44	III	EC_1B	ectopic stromal cells
			EU_1A	eutopic stromal cells
2	29	III	EC_4B	ectopic stromal cells
			EU_4A	eutopic stromal cells
3	31	IV	EC_19009	ectopic stromal cells
			EU_19008	eutopic stromal cells
4	45	III	EN19	ectopic stromal cells
			EN18	eutopic stromal cells

Table S2. Top 10 differentially expressed upregulated and downregulated mRNAs with the highest fold change.

**Gene symbol**	**Gene name**	**Log_2 fold change**	**FDR**
Upregulated in ectopic endometrial stromal cells			
SEC14L4	SEC14 like lipid binding 4	10.9	2E-04
SNAI3	snail family transcriptional repressor 3	10.3	1E-03
CFAP99	cilia and flagella associated protein 99	10.1	2E-02
CACNA1B	calcium voltage-gated channel subunit alpha1 B	8.6	6E-04
VIPR1	vasoactive intestinal peptide receptor 1	6.8	6E-05
DNER	delta/notch like EGF repeat containing	6.3	8E-04
CABP1	calcium binding protein 1	6.1	3E-03
PAQR9	progestin and adipoQ receptor family member 9	5.8	8E-04
SLC22A3	solute carrier family 22 member 3	5.7	2E-02
VSIG1	V-set and immunoglobulin domain containing 1	5.4	1E-02
Downregulated in ectopic endometrial stromal cells			
MYL3	myosin light chain 3	− 14.3	4E-05
GLRA4	glycine receptor alpha 4	− 10.6	3E-02
MAPK4	mitogen-activated protein kinase 4	− 9.2	1E-02
ADCYAP1R1	ADCYAP receptor type I	− 8.5	3E-02
HOXD13	homeobox D13	− 5.8	1E-05
KIAA1456	KIAA1456	− 4.3	7E-05
FOXD2	forkhead box D2	− 3.2	2E-06
MYPN	myopalladin	− 3.2	1E-02
CGA	glycoprotein hormones, alpha polypeptide	− 3.1	2E-02
RNF182	ring finger protein 182	− 3.1	1E-02

Table S3. KEGG analysis showed that the DEGs were mostly involved in three pathways: the PI3K-Akt signaling pathway, cytokine-cytokine receptor interaction, and MAPK signaling pathway.

Category	n	Involved DEGs
PI3K-Akt signaling pathway	39	IGF1, TNC, LAMC2, NGFR, FGF22, PPP2R2C, COL4A4, ITGA6, FLT1, ITGB8, MET, CSF3, AREG, VEGFA, GHR, CCND2, PGF, KDR, ITGA7, IL6, TLR2, FGF7, COL6A1, COL6A2, CREB5, FGF18, IL6R, ITGA5, PRKAA2, TGFA, NOS3, DDIT4, GNG4, IL7R, COL4A3, INSR, NTF3, GNG2, LPAR1
Cytokine-cytokine receptor interaction	36	IL32, TNFRSF1B, NGFR, CXCL2, TGFB2, TGFBR1, CSF3, PF4V1, GHR, IL1A, CCL20, CXCR4, INHBA, CXCL6, IL1B, GDF5, LIF, IL6, IL1RN, IL33, TSLP, TNFRSF21, CCL28, GDF6, EDA, IL6R, CCR5, IL24, CXCL3, CXCL5, CXCL1, IL15, CSF2, IL7R, CXCL8, IFNE
MAPK signaling pathway	32	MAP3K14, CACNA1G, IGF1, RASGRF1, NGFR, FGF22, CACNG4, TGFB2, FLT1, CACNG7, MET, TGFBR1, MAPK8, MAP2K6, AREG, PTPN5, VEGFA, RASGRF2, IL1A, STMN1, PGF, DUSP4, IL1B, KDR, NFATC1, FGF7, FGF18, TGFA, CD14, INSR, NTF3, PLA2G4E

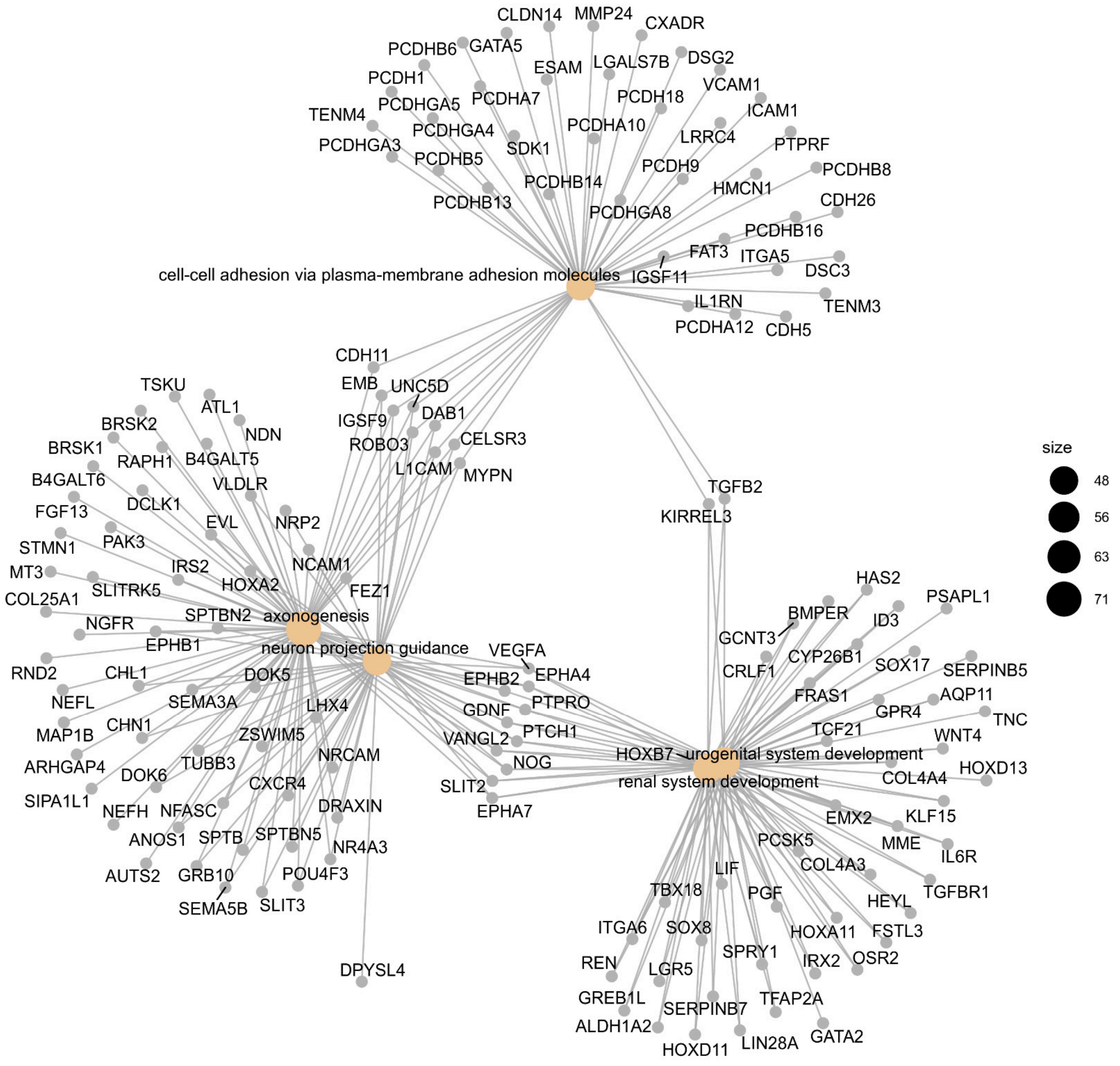

Figure S1. The genes associated with the five GO biological processes terms are illustrated by the cnetplot.

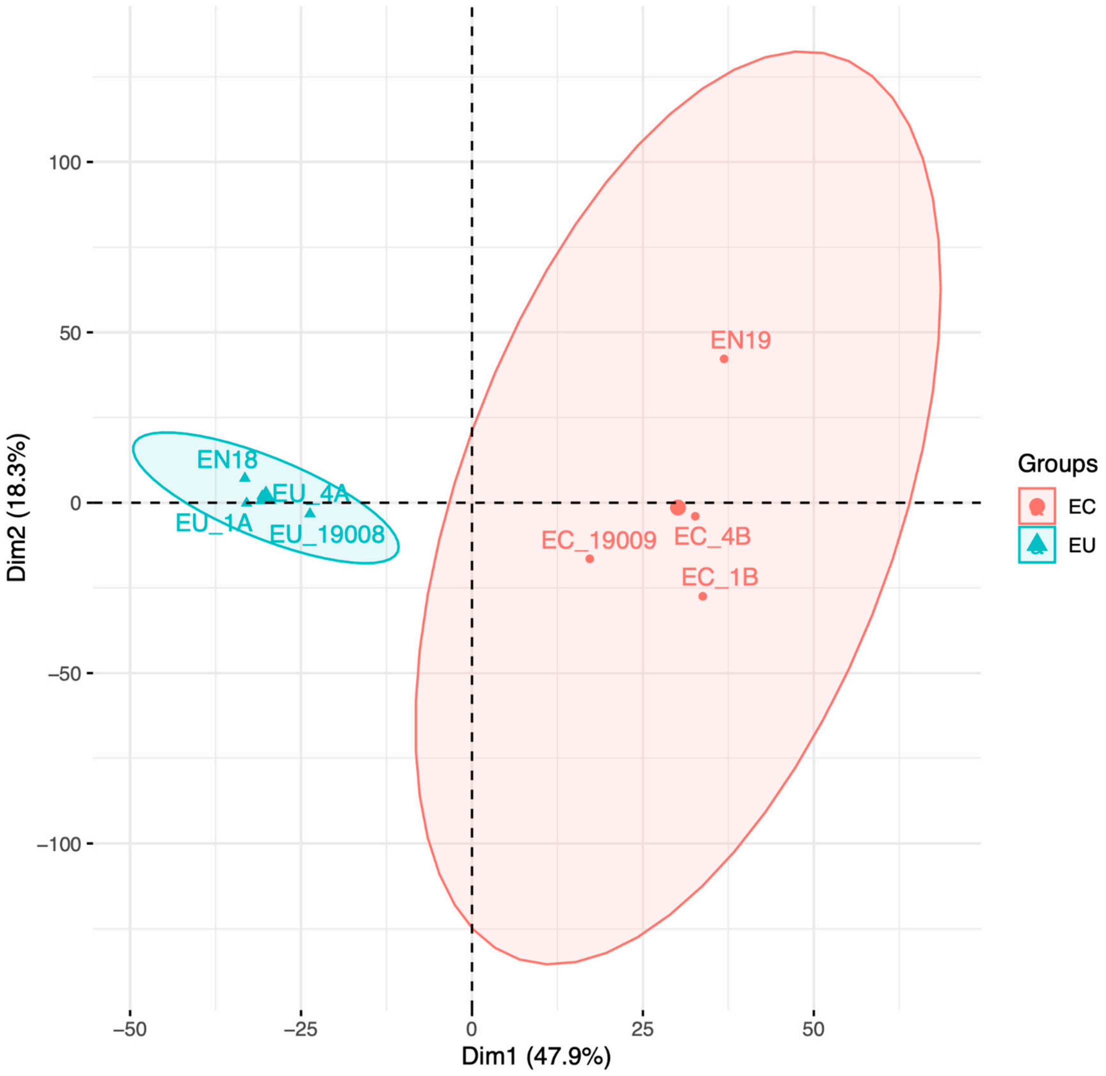

Figure S2. PCA plot of RNA-seq data show the characteristics of samples according to gene expression (FPKM) levels. Each dot indicates a sample.
